# Australian and New Zealand Glaucoma Society (ANZGS) Consensus on Interventional Glaucoma

**DOI:** 10.1111/ceo.70140

**Published:** 2026-07-06

**Authors:** Graham A. Lee, Lance Liu, Ridia Lim, Rajiv Shah, Robert J. Casson, Keith R. Martin, Helen Danesh‐Meyer, Ashish Agar, Jennifer C. Fan Gaskin, Judy Y. Ku, Geoffrey Chan, Anna Galanopoulos, Shamira Perera

**Affiliations:** ^1^ Queensland Academy of Ophthalmology, Queensland Children's Hospital Brisbane Australia; ^2^ The Victorian Eye & Ear Hospital Melbourne Australia; ^3^ Sydney Eye Hospital Sydney Australia; ^4^ Bankstown Public Hospital Bankstown Australia; ^5^ Adelaide University Adelaide Australia; ^6^ University of Auckland Auckland New Zealand; ^7^ University of New South Wales Sydney Australia; ^8^ Queensland University of Technology Brisbane Australia; ^9^ Lions Eye Institute Perth Australia; ^10^ Royal Adelaide Hospital Adelaide Australia; ^11^ Singapore National Eye Centre Singapore

**Keywords:** disease progression, glaucoma, intraocular pressure, laser therapy, minimally invasive surgical procedures, practice guideline

## Abstract

**Background:**

Traditional reactive ‘wait and see’ approaches in glaucoma management often result in unacceptable rates of permanent vision loss. This consensus defines a proactive ‘interventional glaucoma’ framework tailored for the Australian and New Zealand healthcare context.

**Methods:**

A modified Delphi consensus process was utilised, consisting of a lead panel of 12 experts (10 Australian, 1 New Zealand, and 1 Singaporean) and a validation cohort of 55 glaucoma specialists and ophthalmologists. Consensus was established via live panel presentations and real‐time electronic polling. Participants evaluated statements using a three‐tier scale: agree, stand aside, or object, with a pre‐defined consensus threshold of an objection rate < 20%.

**Results:**

Twelve consensus statements were ratified. Key points include the formalised classification of glaucoma as a progressive optic neuropathy and the staging of disease to personalise target intraocular pressures to minimise progression. Selective laser trabeculoplasty and Minimally Invasive Glaucoma Surgery were recognised for their role in reducing the daily medication burden for mild‐to‐moderate disease. Traditional incisional surgeries remain the gold standard for advanced or refractory cases. The emerging role of Polygenic Risk Scores in adult‐onset glaucoma was also acknowledged.

**Conclusions:**

This Australian and New Zealand Glaucoma Society consensus on interventional glaucoma advocates for a shift towards an interventional paradigm. By utilising advanced diagnostics and timely procedural interventions, clinicians can better preserve visual function and optimise long‐term patient quality of life.

## 1 Introduction

The concept of interventional glaucoma indicates a paradigm shift in ophthalmic practice, moving from a traditional stepwise approach to a proactive, procedure‐oriented mindset [1]. This philosophy prioritises early intervention, advanced diagnostics, laser therapies like Selective Laser Trabeculoplasty (SLT) and Minimally Invasive Glaucoma Surgery (MIGS) to stabilise intraocular pressure (IOP) and reduce the need for lifelong topical medication adherence. However, while the core tenets of interventional glaucoma have commonalities, the practical application of this framework must be tailored to specific regional contexts. Significant disparities in healthcare infrastructure, varying access to the latest surgical devices, and the availability of diverse pharmaceutical options necessitate a localised approach. By adapting these general principles to the available regional resources, clinicians can translate the interventional mindset into equitable and effective care that addresses the unique needs of their patient populations.

The interventional paradigm has been published as international consensus statements and treatment protocols. Funke et al. published a framework for interventional glaucoma across all disease stages, from ocular hypertension to severe neuropathy based on the current availability of laser, procedural pharmaceutics and surgical devices in the United States [2]. The LiGHT trial results from the United Kingdom, have redefined global standards, with organisations such as the European Glaucoma Society (EGS) and the National Institute for Health and Care Excellence (NICE) now endorsing SLT as a first‐line therapy [3].

The current document represents the first interventional glaucoma consensus developed by specialists from the Australian and New Zealand Glaucoma Society (ANZGS), specifically targeting the management of glaucoma patients in this region. This document is intended as a pragmatic expert consensus framework informed by the available evidence and contemporary clinical practice, rather than a formal evidence‐graded clinical guideline. While many recommendations are supported by established clinical trial data and published literature, others reflect expert interpretation in areas where comparative evidence remains incomplete or evolving. Accordingly, these recommendations should be applied in the context of individual patient circumstances, clinician judgement, and emerging evidence.

## 2 Methods

This consensus was developed and reported in accordance with the ACCORD (Reporting Guideline for Biomedical Consensus Methods) statement. The study utilised a modified Delphi process to establish a proactive framework for interventional glaucoma management. The consensus process involved a lead panel of 12 experts, comprising 10 specialists from Australia, one from New Zealand, and one from Singapore. The findings were further validated by a broader cohort of 55 glaucoma specialists and ophthalmologists with a dedicated interest in the field. The core topics and initial draft statements were identified through a comprehensive review of established clinical trials, international consensus statements, and regional treatment protocols. Draft recommendations were developed by integrating this evidence base with expert clinical interpretation relevant to Australian and New Zealand practice.

Consensus was established via a multi‐stage iterative process:

- *Initial polling*: Following the live presentations, statements were evaluated by the lead panel and validation cohort using real‐time electronic polling
- *Voting scale*: Participants used a three‐tier scale: agree, stand aside (indicating minor reservations without formal opposition) or object.
- *Iterative refinement*: Following the live session, statements were further refined through a series of face‐to‐face and virtual discussions involving lead authors (G.A.L., L.L., R.L., R.S., R.J.C., K.R.M., H.D.M., S.P.) and individual panel members to address specific feedback and nuances in the phrasing.
- *Consensus threshold*: In accordance with the pre‐defined protocol, consensus for each statement was defined as an objection rate of less than 20%. All 12 final statements met this threshold.
- This methodology reflects a consensus‐building process rather than a formal systematic evidence grading exercise. Accordingly, the resulting recommendations should be interpreted as expert consensus statements informed by available evidence rather than prescriptive evidence‐graded practice guidelines.

### 2.1 Ethics and Data Protection

As this research was limited to a structured consensus‐building process among healthcare professionals and did not involve patient participation, clinical interventions, or the use of patient‐identifiable data, formal institutional ethics committee review was not sought. The study was conducted in adherence to the tenets of the Declaration of Helsinki. Participant names and affiliations were used solely for the operations of the publication in accordance with data protection policies.

## 3 Results

### 3.1 Consensus Statement #1

Interventional glaucoma is a proactive philosophical shift in disease management—moving away from a traditional stepwise approach—that emphasises earlier, targeted interventions to prevent vision loss and improve the patient's overall quality of life [1].

### 3.2 Reasoning

- *Unacceptable rates of blindness*: The panel noted that traditional management strategies still result in too many patients going blind. Data from a study of 592 open‐angle glaucoma patients in Malmo, Sweden, demonstrated a 42% lifetime risk of going blind in at least one eye, with a 25% cumulative incidence of blindness at 10 years and 40% at 20 years [4]. More conservative estimates place the risk of blindness at 15% at 15 years after diagnosis, a rate the panel deemed ‘far too high’ [5].
- *Proactive vs. reactive care*: Historically, glaucoma management has been reactive, often delaying significant intervention until the patient begins to lose sight
- *Reducing the medication burden*: A major aim is to reduce the patient's burden of daily topical medications. Topical drops are associated with poor patient adherence and adverse side effects, which negatively impact outcomes.
- *Tailored and timely treatment*: The panel agreed that ‘interventional’ does not necessarily mean performing new surgeries just because the technology exists; rather, it provides a framework to individualise which intervention is most appropriate for that patient and when they need it to reduce the risk of visual field progression.
- *Long‐term quality of life*: By reducing the medication burden and intervening earlier to stabilise the disease, clinicians can help patients decrease the need for major, higher‐risk incisional surgeries later in their lives, ultimately reducing patient morbidity and improving their quality of life.

### 3.3 Consensus Statement #2

Glaucoma is defined as a progressive optic neuropathy characterised by the degeneration of retinal ganglion cells, which results in specific structural damage to the optic nerve and/or retinal nerve fibre layer and associated detection of loss of visual function [6].

### 3.4 Reasoning

- *A paradigm shift*: Historically, the medical community equated glaucoma directly with high IOP. The current consensus, however, reclassifies it as a neurodegenerative disease of the optic nerve.
- *The role of eye pressure*: While elevated IOP is the most significant modifiable risk factor for developing the disease, it is no longer the defining diagnostic criterion. This is because patients can suffer glaucomatous nerve damage despite having statistically ‘normal’ eye pressures (normal‐tension glaucoma), while others may have high pressures without ever developing nerve damage (ocular hypertension).
- *Structural damage*: Generally precedes functional loss: the disease process is often first identified through structural alterations, including characteristic thinning of the neural retinal rim and retinal nerve fibre layer. While retinal ganglion cell dysfunction may precede irreversible tissue loss, structural imaging is often more sensitive than standard automated perimetry for detecting early glaucomatous change, as visual field testing is inherently more variable, sparsely samples the visual field, and uses a logarithmic decibel scale that can reduce sensitivity to early functional loss. Corresponding functional deficits may therefore only become apparent later as characteristic visual field defects emerge.
- *Diagnostic focus*: Because relying solely on IOP measurements is inadequate, the panel emphasises that a definitive diagnosis requires careful, direct clinical examination and imaging of the optic nerve head, alongside functional visual field testing.

### 3.5 Consensus Statement #3

The formal staging of glaucoma severity is a critical step in clinical management, as it guides the individualised target IOP, determines the frequency of clinical monitoring, and informs the appropriateness of therapeutic and surgical interventions. The therapeutic objective in glaucoma is not simply maximal IOP reduction, but optimisation of cumulative lifetime quality of life by balancing glaucoma‐related morbidity against treatment‐related morbidity. Accordingly, target IOP should be understood as an individualised therapeutic construct rather than a fixed numerical threshold [6].

### 3.6 Reasoning

- *Standardised assessment*: Accurately grading severity requires synthesising both functional visual loss and structural damage. Clinicians typically rely on standardised classification systems (such as the Hodapp–Parrish–Anderson [7] and Glauc‐Strat Fast [8]), which use visual field indices—like Mean Deviation (MD) and the proximity of defects to the central point of fixation—alongside patient factors including co‐morbidities and socio‐economic status.
- *Target IOP stratification*: The panel agreed on pragmatic consensus target ranges informed by landmark glaucoma trials, established clinical practice patterns, and expert clinical experience. These targets are intended as practical clinical guides rather than rigid evidence‐based thresholds and should be individualised according to untreated baseline IOP, documented rate of progression, patient age, life expectancy, co‐morbidities, and other risk factors.
  ○ *Mild glaucoma*: May safely tolerate a target IOP of ≤ 18 mmHg and a reduction of ≥ 20% from baseline.
  ○ *Moderate glaucoma*: May safely tolerate a target IOP of ≤ 15 mmHg and a reduction of ≥ 30% from baseline
  ○ *Severe/advanced glaucoma*: Has a narrower margin of safety. It requires a much more aggressive target IOP ≤ 12 mmHg (and potentially single digits) and a reduction of ≥ 40% from baseline in order to preserve the remaining retinal ganglion cells and protect the central island of vision.
- *Quality of life and functional independence*: The panel emphasised that severity directly correlates with the patient's real‐world functional impairment. Patients with advanced visual field loss face significant challenges with mobility, driving, and reading. Because the risk of total blindness is higher, these patients require closer surveillance and shorter intervals between follow‐up appointments.
- *Threshold for intervention*: A patient's severity grade alters the risk–benefit calculus of surgery. While mild or moderate disease might be managed conservatively or with Minimally Invasive Glaucoma Surgery (MIGS), severe disease with documented progression often warrants bypassing stepwise therapies in favour of immediate, more effective incisional surgeries (such as a trabeculectomy or glaucoma drainage devices) to secure a low target IOP as soon as possible (Figure 1).

**FIGURE 1:**
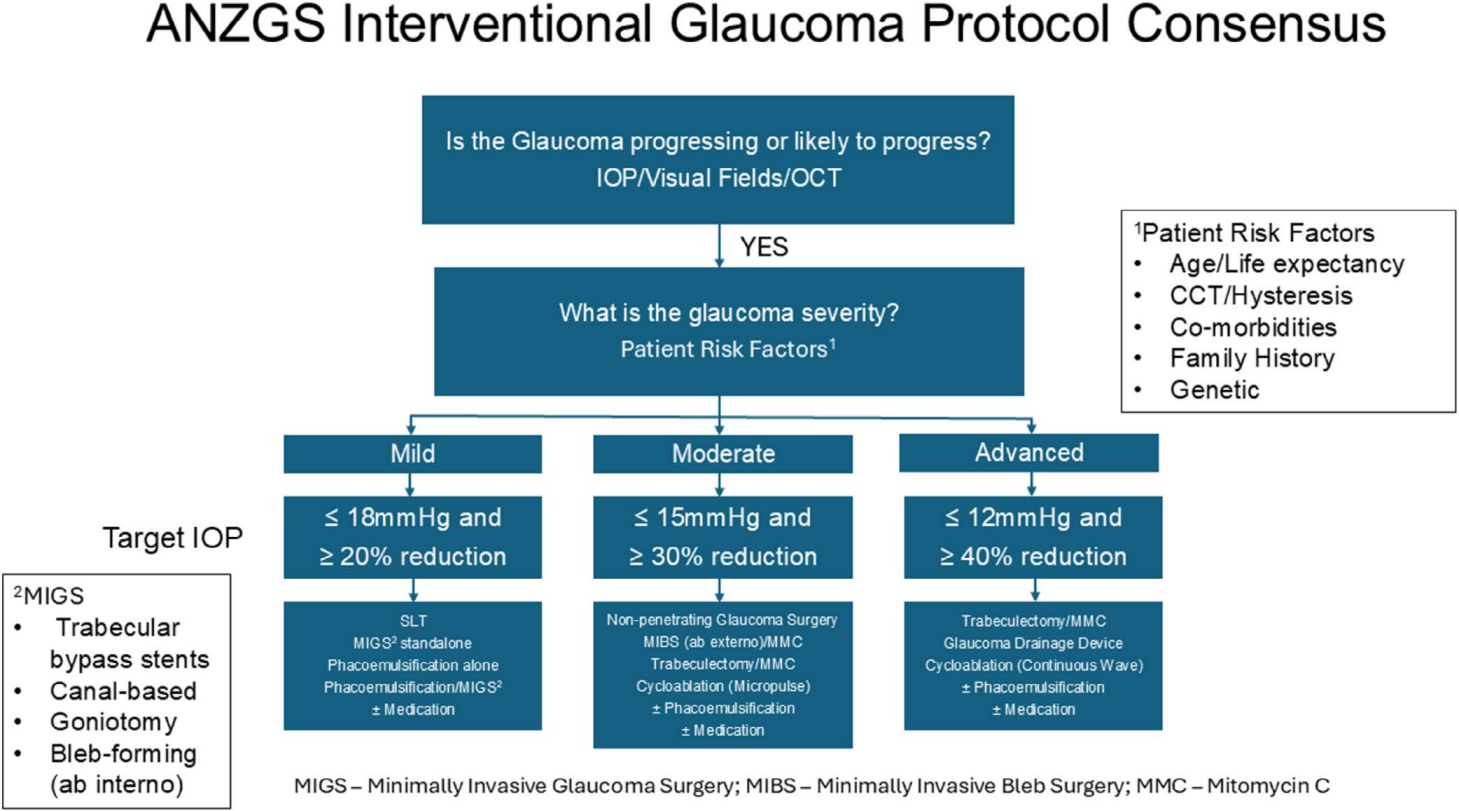
ANZGS consensus framework for interventional glaucoma management.

### 3.7 Consensus Statement #4

IOP remains the sole proven, modifiable risk factor for the development and progression of glaucoma; however, comprehensive management must evolve beyond single, static clinic measurements to account for dynamic diurnal fluctuations, peak pressures, and individual corneal biomechanics to establish customised target pressures [9].

### 3.8 Reasoning

- *The modifiable target*: While glaucoma is fundamentally a neurodegenerative optic neuropathy, mechanical and vascular theories both point to IOP as the primary driver of strain on the lamina cribrosa. Lowering IOP is currently the only evidence‐based intervention proven by major trials (like the AGIS [10] and EMGT [11]) to halt or significantly slow retinal ganglion cell apoptosis and preserve the visual field.
- *Fluctuation and peak IOP*: Relying strictly on snapshot, office‐hour measurements is insufficient. Clinical consensus increasingly recognises that nocturnal pressure peaks and wide diurnal fluctuations are strong, independent predictors of disease progression. Patients experiencing significant circadian IOP swings (such as demonstrated by serial IOP measurements at different times or indicated by a water drinking test) often have continued structural damage even when their ‘average’ clinic IOP appears adequately controlled.
- *The impact of corneal biomechanics*: Standard Goldmann applanation tonometry is inherently confounded by the structural properties of the cornea. Central corneal thickness can artefactually inflate or deflate readings, but more importantly, corneal hysteresis serves as an independent risk factor. A low hysteresis indicates a reduced biomechanical buffering capacity of the ocular tissues, directly correlating with an increased vulnerability to optic nerve damage at any given IOP.
- *Dynamic target IOP*: Setting a target pressure is a continuous, dynamic process rather than a one‐time calculation. It needs to factor in the severity of the disease, the rate of progression, and the patient's life expectancy. Particularly when managing complex or advanced cases, the target must frequently be titrated down to achieve disease stability.

### 3.9 Consensus Statement #5

Genetic testing in glaucoma has clinical utility for early‐onset, strongly familial, and syndromic forms of the disease, where it guides targeted cascade screening and early intervention; meanwhile, its application in typical adult‐onset Primary Open‐Angle Glaucoma (POAG) is progressively transitioning from a research tool to clinical practice via the development of Polygenic Risk Scores (PRS) [12].

### 3.10 Reasoning

- *High‐penetrance mutations*: In cases of Juvenile Open‐Angle Glaucoma (JOAG) or Primary Congenital Glaucoma (PCG), single‐gene mutations (such as in the MYOC, CYP1B1 or OPTN genes) play a dominant role. Identifying a specific, highly penetrant mutation allows clinicians to definitively diagnose the underlying cause and predict the aggressiveness of the disease.
- *Cascade screening for relatives*: The most immediate benefit of identifying a high‐risk mutation in a patient (the proband) is the ability to offer targeted genetic testing to their asymptomatic family members. Relatives who test higher‐risk can be placed on a regular surveillance programme to catch and treat elevated IOP to prevent irreversible optic nerve damage, while those who test lower‐risk can be spared unnecessary lifelong anxiety and aggressive screening.
- *The polygenic challenge of POAG*: Unlike early‐onset forms, standard adult‐onset POAG is a complex, polygenic trait. This means it is caused by the cumulative, tiny effects of hundreds or thousands of different genetic variants, combined with environmental factors. Therefore, testing for a single gene is unhelpful for most adult glaucoma patients.
- *The emergence of PRS*: To address the polygenic nature of POAG, the field is moving towards PRS. By analysing a wide array of genetic markers simultaneously, a PRS can stratify the general population into risk tiers. Patients in the highest genetic risk percentiles are more likely to develop advanced disease and require surgical intervention, making PRS a promising future tool for personalised screening protocols.
- *Requirement for ophthalmic consultation*: The panel highlights that genetic testing must always be paired with ophthalmic consultation. The results can carry significant psychological weight and potential implications for life or health insurance, so patients must fully understand the risks, benefits, and limitations of the information before proceeding.

### 3.11 Consensus Statement #6

Accurately identifying glaucomatous progression requires a comprehensive, longitudinal approach that integrates both structural imaging and functional visual testing, utilising both event‐based and trend‐based analyses to distinguish true pathological deterioration from normal age‐related changes and long‐term testing variability [6].

### 3.12 Reasoning

- *Structure vs. function disconnect*: Glaucomatous damage does not always present simultaneously across different diagnostic modalities. In early‐stage glaucoma, structural changes—such as thinning of the Retinal Nerve Fibre Layer (RNFL) or Macular Ganglion Cell Complex (GCC)—often precede detectable visual field loss. Conversely, in advanced disease, structural imaging reaches a ‘floor effect’ where further tissue loss cannot be reliably measured, making functional visual field testing the primary indicator of ongoing progression.
- *Event‐based vs. trend‐based analysis*: Identifying progression relies on two distinct analytical methods:
  ○ *Event‐based analysis* compares a current test to an established baseline to see if a specific threshold of change has been crossed (e.g., ‘Has a new defect appeared?’).
  ○ *Trend‐based analysis* evaluates multiple tests over time using linear regression to calculate the rate of change (e.g., ‘How rapidly is the visual field deteriorating in decibels per year?’). This rate is critical for predicting whether a patient is at risk of functional blindness during their expected lifetime.
- *Managing testing ‘noise’*: Visual field testing is inherently subjective and prone to significant fluctuation due to patient fatigue, learning curves, or simple day‐to‐day variability. A single worse result is rarely sufficient to declare progression; clinical consensus dictates that suspected progression must be confirmed with subsequent, repeated testing. Establishing a robust baseline with multiple reliable tests early in the diagnosis is essential
- *Accounting for confounders*: Clinicians must be vigilant in ruling out non‐glaucomatous causes for structural or functional worsening. The development of cataracts, severe dry eye disease, macular degeneration, or imaging artefacts (like poor signal strength on an OCT) can mimic glaucomatous progression and lead to unnecessary escalations in therapy if not carefully identified

### 3.13 Consensus Statement #7

Topical IOP‐lowering medications remain a highly effective modality for glaucoma management; however, long‐term success requires carefully balancing the therapeutic efficacy of these drugs against the significant real‐world challenges of patient non‐adherence, systemic side effects, and cumulative ocular surface toxicity.

### 3.14 Reasoning

- *Mechanisms of action*: Medical therapy targets the fundamental fluid dynamics of the eye to lower IOP. Medications broadly fall into two categories: those that suppress the production of aqueous humour in the ciliary body (such as beta‐blockers, alpha‐agonists, and carbonic anhydrase inhibitors) and those that facilitate greater fluid outflow through the trabecular meshwork or uveoscleral pathways (such as prostaglandin analogues, rho‐kinase inhibitors, and miotics).
- *First‐line standard*: Prostaglandin analogues are generally considered the first‐line medical therapy. Their once‐daily dosing profile at night‐time, combined with superior efficacy and an absence of systemic cardiopulmonary side effects, makes them the most favourable starting point for newly diagnosed patients.
- *The adherence hurdle*: The clinical consensus acknowledges that the greatest weakness of medical therapy is not the pharmacology but patient adherence. Complex, multi‐drop regimens, physical difficulty in aiming and squeezing the bottles (especially for elderly patients with arthritis), and forgetfulness frequently lead to unchecked IOP spikes and silent disease progression.
- *Ocular surface disease (OSD) and preservatives*: Chronic, lifelong use of topical drops—particularly those utilising preservatives like benzalkonium chloride (BAK)—inevitably damages the corneal epithelium and conjunctiva. Severe OSD not only causes chronic discomfort and drives down patient adherence, but the resulting conjunctival inflammation can compromise the success rates of any future incisional glaucoma surgeries (like trabeculectomies).
- *The shifting paradigm*: Because of the compounding issues of adherence and OSD, the role of daily medications is slowly shifting. While still critical, clinicians are increasingly utilising preservative‐free formulations, fixed‐combination drops (to reduce the total number of instillations), early SLT, and emerging sustained‐release drug delivery implants to actively minimise the patient's daily medication burden.

### 3.15 Consensus Statement #8

Selective Laser Trabeculoplasty (SLT) can be an effective, safe, and cost‐efficient first‐line option to treat primary open‐angle glaucoma and ocular hypertension, avoiding the challenges of medication non‐adherence.

### 3.16 Reasoning

- *Targeted mechanism of action*: SLT utilises a Q‐switched, frequency‐doubled Nd:YAG laser (532 nm) to selectively target intracellular melanin within the trabecular meshwork endothelial cells. Crucially, its ultra‐short pulse duration (3 ns) limits the thermal diffusion to surrounding non‐pigmented tissues, remaining below the threshold for coagulative necrosis. Rather than causing mechanical scarring, SLT triggers a localised biochemical cascade. This cellular signalling recruits macrophages to clear cellular debris and remodel the extracellular matrix, reducing resistance and improving aqueous outflow.
- *The LiGHT trial paradigm shift*: The clinical consensus has been heavily shaped by landmark studies like the LiGHT (Laser in Glaucoma and Ocular Hypertension) trial [13]. The evidence demonstrates that using SLT as an initial therapy provides equivalent or superior IOP control compared to starting with topical drops. Patients in this trial treated with primary SLT were shown to achieve long‐term disease stability and are less likely to require incisional glaucoma surgeries. Of note, however, large, real‐world studies have failed to achieve similar efficacy.
- *Eliminating the adherence variable*: The potential real‐world advantage of SLT is that it reduces patient adherence from the equation. It provides 24/7, continuous IOP lowering without relying on the patient to remember their daily drops, to instil them correctly, or afford recurring prescription costs [14].
- *Preserving the ocular surface*: By delaying or eliminating the need for daily topical medications, SLT protects the eye from chronic exposure to preservatives like BAK. Preventing medication‐induced OSD improves patient comfort and preserves the health of the conjunctiva, which is important for future glaucoma filtering surgery.
- *Repeatability*: Because SLT does not cause the coagulative necrosis or structural scarring seen with older technologies like Argon Laser Trabeculoplasty (ALT), the trabecular architecture remains intact. This makes SLT a repeatable procedure if the IOP‐lowering effect naturally wanes over a few years, although there is limited efficacy after > 2 procedures [14].
- It was acknowledged by the panel that not all patients are willing to undergo SLT as primary management of their glaucoma and prefer eye drop therapy. There may be various reasons for this decision including the cost of the laser compared to eye drops and fear of ‘laser’ as potentially more risky. This may also have a cultural bias. It was also recognised that SLT is not always effective and that real‐world data may not reflect efficacy attained in clinical trials.

### 3.17 Consensus Statement #9

Minimally Invasive Glaucoma Surgery (MIGS) in the management of mild–moderate glaucoma offers a safer alternative to traditional filtering surgeries that can lower IOP and reduce the daily medication burden, particularly when performed concurrently with cataract extraction [15].

### 3.18 Reasoning

- *Bridging the treatment gap*: Historically, surgeons faced a therapeutic void between conservative therapies (topical drops and SLT) and more invasive, higher risk procedures (like trabeculectomy or tube shunts). MIGS is an option for mild–moderate glaucoma as the IOP lowering is often limited to 2–5 mmHg; greater IOP lowering for more severe glaucoma generally requires other, more invasive surgery.
- *Enhancing physiological outflow*: The majority of commonly deployed MIGS procedures lower intraocular pressure by targeting the trabecular meshwork, the primary site of pathological resistance. By stenting open, mechanically dilating, or excising the trabecular meshwork to access Schlemm's canal, these devices optimise the eye's conventional, physiological aqueous outflow pathway.
- *Favourable safety profile and rapid recovery*: Trabecular bypass stents, canal‐based viscodilation and goniotomy procedures utilise an ab interno, micro‐incisional approach. This approach preserves the conjunctival and scleral tissues, thereby avoiding the creation of a surgical bleb and its associated long‐term complications. Bleb‐forming micro‐invasive procedures, while less invasive than traditional filtering surgeries like trabeculectomy, rely on transscleral filtration into the subconjunctival space, necessitating the use of intraoperative anti‐metabolites and sharing the inherent risks of bleb‐related complications.
- *Synergy with phacoemulsification*: MIGS procedures can be performed with cataract surgery. Because standalone phacoemulsification often exerts only a limited and unpredictable IOP‐lowering effect, combining a MIGS procedure into the same operative session can result in a compounding hypotensive effect without appreciably extending surgical time or prolonging the patient's postoperative recovery period.
- *Medication independence and quality of life*: The goal of MIGS in mild–moderate glaucoma is to reduce the IOP and/or reduce the reliance on drops. This aims to improve the patient's OSD and quality of life.

### 3.19 Consensus Statement #10

Incisional drainage surgeries, specifically trabeculectomy and glaucoma drainage devices, remain the gold standard for lowering IOP in the management of high‐pressure glaucoma, advanced, progressive, or refractory glaucoma, when other options are not sufficient [16, 17].

### 3.20 Reasoning

- *Trabeculectomy*: Incisional drainage surgery aims to form an external fluid reservoir (bleb) that bypasses the eye's natural drainage pathway to achieve low target IOP. The utilisation of an *ab externo* microshunt to replace the sclerostomy can achieve moderate short‐term IOP reduction; however, its long‐term efficacy is typically inferior to a standard trabeculectomy. Similarly, non‐penetrating glaucoma surgery such as deep sclerectomy or viscocanalostomy can achieve moderate pressure lowering with a reduced risk of early postoperative hypotony. Nevertheless, these non‐penetrating modalities may eventually require conversion to a penetrating procedure via Nd:YAG laser goniopuncture to achieve lower target IOP.
- *Wound healing modulation*: The primary cause of failure in filtration surgery is the subconjunctival wound healing response, which attempts to scar down and occlude the newly created drainage pathway. The intraoperative application of anti‐metabolites, such as mitomycin C (MMC) or 5‐fluorouracil (5‐FU), is routinely employed to suppress fibroblast proliferation and minimise scarring. The specific concentration, exposure duration, and choice of agent remain dependent on surgeon preference, the patient's clinical risk profile, and tissue characteristics.
- *Glaucoma drainage devices*: Commonly referred to as tube surgery, these drainage devices are indicated when a trabeculectomy is considered less likely to succeed due to an elevated risk of an aggressive wound healing response. Classic indications include pre‐existing conjunctival scarring (e.g., from prior ocular surgery), neovascular glaucoma, paediatric glaucoma and uveitic or inflammatory glaucoma.
- *Intensive postoperative management*: Because incisional and device‐based procedures fundamentally alter the structural integrity of the eye and carry a more significant risk profile than conservative therapies, they demand intensive, individualised postoperative monitoring. Vigilant assessment allows for timely, early optimisation of long‐term surgical success.

### 3.21 Consensus Statement #11

Cycloablation is traditionally reserved for end‐stage or complex glaucoma cases; however, it may be considered at an earlier stage of disease as a bridge to planned drainage surgery, or as a standalone alternative when invasive filtering surgery is not a viable option.

### 3.22 Reasoning

- *Mechanism of action*: Cycloablative procedures lower IOP by targeting the ciliary body epithelium, thereby decreasing the production of aqueous humour.
- *Continuous‐wave*: Continuous‐wave transscleral cyclophotocoagulation (CW‐TSCPC) utilises a continuous diode laser to induce coagulative necrosis of the ciliary processes. This modality is primarily indicated for the management of painful eyes with refractory glaucoma, as well as cases with poor visual potential or end‐stage disease. However, it may also be utilised in selected cases of less advanced disease, or as a supplementary therapy following tube shunt surgery.
- *Micropulse*: Micropulse transscleral cyclophotocoagulation (MP‐TSCPC) fractionates the laser energy into short, repetitive ‘on’ and ‘off’ intervals (duty cycles). This delivers controlled, sub‐threshold thermal energy designed to minimise collateral tissue damage and limit inflammation while maintaining therapeutic efficacy [18]
- *CW‐TSCPC vs. MP‐TSCPC*: The consensus panel acknowledged that MP‐TSCPC offers a safer alternative to CW‐TSCPC, demonstrating a lower risk of severe complications such as prolonged inflammation, chronic hypotony, and phthisis bulbi. However, MP‐TSCPC typically exhibits lower absolute pressure‐lowering efficacy and a shorter duration of clinical effect than the continuous‐wave modality, often requiring a higher rate of retreatment.

### 3.23 Consensus Statement #12

Postoperative management following glaucoma surgery is an active, therapeutic extension of the operation itself; achieving a successful, long‐term target IOP relies on the control of inflammation, the modulation of wound healing and the proactive identification of both early and late complications [19].

### 3.24 Reasoning

- *Active wound healing modulation*: The host fibrovascular healing response is the primary cause of failure in filtering surgeries. Post‐operative care involves titration of topical corticosteroids alongside timely interventions such as digital ocular massage, suture adjustment, or bleb needling to rescue a failing or encapsulating bleb. For patients identified as having an elevated risk of aggressive subconjunctival scarring, adjunctive anti‐proliferative therapies, such as subconjunctival 5‐FU injections, may be considered.
- *Recognising and treating infection*: While relatively rare, bleb‐related infections including blebitis and late‐onset endophthalmitis remain lifelong risks, particularly in the presence of thin, avascular blebs. Because these conditions are highly sight‐threatening, intensive fortified topical antibiotic therapy must be commenced immediately upon clinical suspicion to prevent rapid intraocular extension.
- *Patient adherence to restrictions*: Long‐term surgical success is heavily dependent on strict patient adherence to both the complex topical steroid regimen and essential behavioural restrictions. Avoiding Valsalva manoeuvres, heavy lifting, vigorous bending, and any eye rubbing during the early post‐operative weeks significantly reduces the risk of structural complications, including over‐drainage, profound hypotony, choroidal effusion, or suprachoroidal haemorrhage.

A flowchart based on the above 12 consensus points is proposed (Figure 1). It is stressed that these are pragmatic consensus guidelines rather than prescriptive evidence‐based universal treatment pathways. Individual patient risk factors must be considered and management regularly reviewed according to clinical response.

## 4 Discussion

This consensus document outlines an interventional paradigm shift in glaucoma management, moving from a reactive ‘wait and see’ approach towards a proactive, interventional philosophy aimed at preventing irreversible vision loss. It defines glaucoma fundamentally as a progressive optic neuropathy, acknowledging that while IOP is the only modifiable risk factor, management requires a comprehensive, longitudinal approach that integrates both structural and functional assessments, formal staging and individualised target pressures. This framework incorporates a wide, tailored spectrum of treatments from medical therapies and SLT to MIGS and glaucoma drainage procedures or cycloablation all while emphasising the need to provide the most appropriate intervention according to the glaucoma severity. The individual patient's needs must be addressed to minimise glaucoma progression and optimise long‐term quality of life.

Within this framework sits a broad, tailored spectrum of treatments ranging from medical therapies and SLT to MIGS, incisional filtration or drainage device surgeries, and cycloablation. Crucially, this philosophy emphasises the necessity of aligning the treatment with the severity of the glaucoma. Ultimately, the unique needs of the individual patient must be addressed to minimise the risk of functional progression while optimising long‐term quality of life.

## Funding

This consensus panel meeting and associated hospitality were supported by an unrestricted educational grant from Alcon. This support was limited strictly to the hosting of the expert panel dinner and logistical arrangements.

## Conflicts of Interest

R.J.C. declares equity in Seonix Pty Ltd. The other authors declare no conflicts of interest.

## Data Availability

The data that support the findings of this study are available on request from the corresponding author. The data are not publicly available due to privacy or ethical restrictions.
